# An efficient multiscale method for subwavelength transient analysis of acoustic metamaterials

**DOI:** 10.1098/rsta.2023.0368

**Published:** 2024-09-23

**Authors:** R. Liupekevicius, J. A. W. van Dommelen, M. G. D. Geers, V. G. Kouznetsova

**Affiliations:** ^1^ Mechanical Engineering, Eindhoven University of Technology, The Netherlands

**Keywords:** locally resonant acoustic metamaterials, homogenization, equivalent fluid model, micromorphic enriched continuum

## Abstract

A reduced-order homogenization framework is proposed, providing a macro-scale-enriched continuum model for locally resonant acoustic metamaterials operating in the subwavelength regime, for both time and frequency domain analyses. The homogenized continuum has a non-standard constitutive model, capturing a metamaterial behaviour such as negative effective bulk modulus, negative effective density and Willis coupling. A suitable reduced space is constructed based on the unit cell response in a steady-state regime and the local resonance regime. A frequency domain numerical example demonstrates the efficiency and suitability of the proposed framework.

This article is part of the theme issue ‘Current developments in elastic and acoustic metamaterials science (Part 2)’.

## 1. Introduction

Locally resonant acoustic metamaterials (LRAMs) exhibit unconventional material behaviour when their response is observed at a macro-scale. These include negative effective mass density (Liu *et al*. [1]), negative effective bulk modulus (Fang *et al*. [2]) and double negativity [3,4]. Local resonance phenomena at the micro-scale constitute the underlying mechanism that triggers such anomalous behaviour and can be tuned to take place in the subwavelength regime, providing compact designs relative to the target wavelength [5]. Typically, when an effective material property becomes negative, acoustic waves cannot propagate, providing an attractive passive solution for wave mitigation, as extensively discussed in Gao *et al*. [6] with promising applications for sound insulation and sound absorption, e.g. aeronautics and building acoustics.

A popular emerging type of LRAM is the so-called labyrinthine metamaterials that owe their name to the induced coiling up of the wave path, first proposed by Liang & Li [7]. The subwavelength character of labyrinthine metamaterials emerges from the space-coiling property, effectively creating a fluid medium with a low effective sound speed relative to the background fluid. When these structures are arranged sparsely, local resonances take place, providing enhanced sound insulation, first demonstrated by Cheng *et al*. [8]. Later, bio-inspired [9] or fractal [10] patterns have been presented, as well as multiple other designs [11–17], just to mention a few. Labyrinthine metamaterials are particularly interesting because they can be easily manufactured, e.g. using three-dimensional printing, with high porosity, leading to efficient, lightweight sound insulators. Additionally, their fluid-based resonance mechanism takes place in the absence of deforming resonant structures, which removes potential fatigue issues.

Metamaterial wave dispersion properties are basically characterized by considering an infinite lattice composed of periodically repeated unit cells. Imposing the so-called Bloch–Floquet periodic boundary conditions allows one to understand the wave propagation behaviour by solving a problem for a single unit cell, thus, at a low computational cost, e.g. see the finite element implementation in Elford *et al*. [18]. Alternatively, the effective material properties can be back-calculated from one-dimensional setups [19–21]. However, the characterization in an infinite domain is rather far from realistic applications where metamaterial devices have three-dimensional boundaries, complex loading conditions and interfaces to other components. Yet, a finite-sized domain problem creates a major concern of computational affordability due to an excessively large number of degrees of freedom required to resolve the full metamaterial structure composed of many unit cells in the time and frequency domain. This identified computational challenge calls for efficient computational methods that enable the full exploration of metamaterial functionalities at the engineering scale, as underlined by Palma *et al*. [22] in the context of integration of metamaterial technology into aeronautic applications.

Dynamic homogenization is a potential solution to these issues and has now achieved a basic level of maturity. For example, the elastodynamic homogenization theory initiated by Willis [23] and extensively revised by Nassar *et al*. [24] and Sridhar *et al*. [25] proves to be exact for an infinite continuum. It defines a macroscopic medium providing macroscopic non-standard constitutive laws for heterogeneous materials. Nonetheless, effective properties in the infinite domain are not necessarily directly transferred to the finite domain, as analysed in Srivastava & Nemat-Nasser [26] and Sridhar *et al*. [27]. In Muhlestein *et al*. [28] and Sieck *et al*. [29], Willis homogenization results from elastodynamics are specific for fluid acoustics, where pressure is a more natural representation of the waves.

Several homogenization methods have been proposed for LRAMs composed of Helmholtz resonators in which the fluid is the wave carrier. Hu *et al*. [30] developed a macroscopic model by deriving analytical formulas of effective properties using a simple cylindrical tree-component geometry unit cell based on a two-step homogenization scheme. Another approach is the rigorous asymptotic homogenization based on scale separation, which has been used to describe wave propagation in rigid porous media with embedded Helmholtz resonators by Boutin [31], and later validated experimentally in Boutin & Becot [32]. Based on ensemble averages, Lafarge & Nemati [33] developed a homogenization method based on acoustics–electromagnetics analogy, providing a rigorous macroscopic description of rigid porous media. The method was capable of describing sound propagation in a sequence of Helmholtz resonators by Nemati *et al*. [34] and in an array of rods in the Bragg scattering regime, i.e. beyond the long wavelength limit [35]. These insightful and physically sound homogenization approaches mainly focus on determining frequency-dependent and wavenumber-dependent material properties of the corresponding macro-scale constitutive models. However, their application to solving finite-size metamaterial problems has not been presented.

Reduced-order computational homogenization, e.g. Sridhar *et al*. [36], is a multiscale technique for linear problems that provides an equivalent macro-scale continuum with non-standard constitutive relations retrieved from microstructural unit cell simulations. It belongs to a class of homogenization methods based on the definition of a representative volume element, as extensively discussed in Geers *et al*. [37,38], and it was first applied to solid metamaterials by Pham *et al*. [39] employing the FE ${,}^{2}$ computational homogenization approach. Reduced-order homogenization allows overcoming computational cost issues, leading to an effective continuum for LRAMs by appropriately transferring the micro-scale inertia to the macro-scale model, which can be straightforwardly implemented, e.g. using the finite element method. Reduced-order computational homogenization provides a macroscopic primary field that approximates the average of the non-resonant domain of the unit cell, and it is based on the so-called relaxed separation of scales, similar to the approach of asymptotic homogenization broadly reviewed in Boutin *et al*. [40]. However, until now, the computational homogenization method has only been applied to elastodynamics, i.e. wave propagation in solid metamaterials described by means of a displacement-based formulation. In contrast, the pressure field naturally describes metamaterials relying on fluid resonance, such as labyrinthine metamaterials, revealing the need for a pressure-based framework.

In this article, we propose an efficient macro-scale model for acoustic metamaterials in which pressure is the primary field, as is typically the case in acoustics. The present work provides an enriched continuum capable of capturing negative effective properties and Willis coupling. It enables assessing the performance of metamaterials beyond the conventional infinite domain restriction for both transient and time-harmonic wave propagation problems.

The article is organized as follows: first, the governing equations of the macro-scale problem are defined in §2. Second, the unit cell governing equations and the model reduction based on subwavelength resonances are elaborated in §3a,b, respectively. Next, the computational homogenization framework is presented by coupling macro- and micro-scales in a variationally consistent manner based on the extended Hill–Mandel condition in §3c, followed by the development of an enriched continuum that describes LRAMs in §3d. Finally, numerical examples show the comparison between the full-field response and the response of the proposed homogenized continuum in infinite and finite domains in §4. Conclusions are given in §5.

For clarity, the mathematical notation is defined in this paragraph. The material derivative is equivalent to the spatial time-derivative due to the use of linearized equations for the pressure fluctuations in the absence of mean flow, see Goldstein [41]; thus, they can be used interchangeably as $\frac{\partial }{\partial t}\left(\;\;\;\right)\equiv \overset{˙}{\left(\;\;\;\right)}$ . The mathematical objects used are scalars (zeroth-order tensor), vectors (first-order tensor) and second-order tensors and are represented as $a,\vec{a}$ and 𝐀 , respectively. The inner product is denoted as $A\cdot B=A_{ij}B_{jk}\vec{e_{i}}⊗\vec{e_{k}}$ , where $\left\{\vec{e_{1}},\vec{e_{2}},\vec{e_{3}}\right\}$ represents the Cartesian basis. The dyadic product is denoted as $\vec{a}⊗\vec{b}=a_{i}a_{j}\vec{e_{i}}⊗\vec{e_{j}}$ , except for the gradient operator where it is omitted, i.e., $\vec{\nabla }\vec{a}=\frac{\partial a_{j}}{\partial x_{i}}\vec{e_{i}}⊗\vec{e_{j}}$ . The second-order unit tensor is $I=\delta_{ij}\vec{e_{i}}⊗\vec{e_{j}}$ , where $\delta_{ij}$ is the Kronecker delta. The inverse and transpose of a second-order tensor are denoted by ${\left(∙\right)}^{-1}$ and ${\left(∙\right)}^{T}$ , respectively. The notations $\left(\underset{∼}{∙}\right)$ and $\left(\underline{∙}\right)$ are used for column and matrix assemblies, respectively. The entries of columns and matrices may be scalars, vectors or tensors. The inverse of a matrix is denoted by ${\left(\;\underline{∙}\;\right)}^{-1}$ . The transpose of a column and a matrix are denoted ${\left(\;\underset{∼}{∙}\;\right)}^{T}$ and ${\left(\;\underline{∙}\;\right)}^{T}$ , respectively. Macroscopic quantities are identified with the subscript ‘ M ’, while microscopic quantities do not have such a subscript.

## 2. Macro-scale homogenized problem

**Figure 1.**
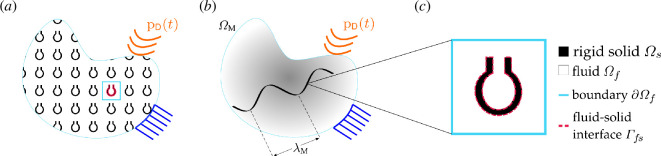
(*a*) Full-field problem. A rigid solid domain (black) and a connected compressible ideal fluid domain (white) define an acoustic metamaterial domain subjected to boundary constraints and time-dependent acoustic loading. (*b*) Equivalent macroscopic domain $\Omega_{\text{M}}$ subjected to boundary constraints and Dirichlet time-dependent acoustic loading $p_{\text{D}}\left(t\right)$ with a representative macroscopic wavelength $\lambda_{\text{M}}$ . (*c*) A unit cell with the definition of the micro-scale rigid solid (black) domain $\Omega_{\text{s}}$ enclosed by fluid–solid interface $\Gamma_{\text{fs}}$ . The fluid (white) domain $\Omega_{\text{f}}$ is enclosed by the surface $\partial \Omega_{f}$ and fluid–solid interface $\Gamma_{fs}$ . The metamaterial unit cell may have resonant cavities such as the depicted Helmholtz resonator.

Consider acoustic wave propagation through a fluid saturating rigid, undeformable channels, a typical problem in acoustics and acoustic metamaterials. The acoustic wave is supported by the fluid, usually air or water. Considering that the numerical simulation of wave propagation through a finite-sized metamaterial, e.g. depicted in figure 1*a*, has prohibitively high computational costs, the reduced-order homogenization technique developed here aims to propose a computationally efficient equivalent macro-scale problem. The idealized effective problem, figure 1*b*, is developed based on the analysis of the response of a micro-scale unit cell, figure 1*c*.

The primary field variable for the acoustic metamaterial case is the macro-scale pressure disturbance $p_{\text{M}}\left(\vec{x_{\text{M}}},t\right)$ (i.e. the pressure disturbances around a quiescent background in the absence of net flow). The relevant macro-scale conservation equation is the acoustic wave equation written in a non-conventional form


(2.1) $$\begin{array}{rl} -{\ddot{\epsilon }}_{M}(\vec{x_{M}},t)+\vec{\nabla_{M}}\cdot {\ddot{\vec{U}}}_{M}(\vec{x_{M}},t) & =0\;\text{in}\;\Omega_{M}\times [t_{1},t_{2}] \end{array}$$

where ${\overset{¨}{\epsilon }}_{\text{M}}$ is the second time-derivative of macro-scale volumetric strain, and ${\overset{¨}{\vec{U}}}_{\text{M}}$ is the macro-scale acceleration. The constitutive models for the conserved quantity ${\overset{¨}{\epsilon }}_{\text{M}}$ and its flux ${\overset{¨}{\vec{U}}}_{\text{M}}$ will be obtained from the upscaling relations of the micro-scale fields, extracting the essential dynamics. In general forms, macro-scale constitutive relations can be written as functions of the macro-scale pressure disturbance $p_{\text{M}}\left(\vec{x_{\text{M}}},t\right)$ , pressure gradient $\vec{\nabla_{\text{M}}}p_{\text{M}}\left(\vec{x_{\text{M}}},t\right)$ and potential ɴ internal enrichment variables $\eta_{j}\left(\vec{x_{\text{M}}},t\right)$ , with j=1,..,ɴ , and their respective time derivatives as


(2.2) $${\ddot{\epsilon }}_{M}={\ddot{\epsilon }}_{M}(p_{M},{\dot{p}}_{M},{\ddot{p}}_{M},\vec{\nabla_{M}}p_{M},\vec{\nabla_{M}}{\dot{p}}_{M},\vec{\nabla_{M}}{\ddot{p}}_{M},\eta_{j},{\dot{\eta }}_{j},{\ddot{\eta }}_{j})\;{\ddot{\vec{U}}}_{M}={\ddot{\vec{U}}}_{M}(p_{M},{\dot{p}}_{M},{\ddot{p}}_{M},\vec{\nabla_{M}}p_{M},\vec{\nabla_{M}}{\dot{p}}_{M},\vec{\nabla_{M}}{\ddot{p}}_{M},\eta_{j},{\dot{\eta }}_{j},{\ddot{\eta }}_{j}).$$

The internal enrichment variables must obey ɴ evolution equations


(2.3) $$g_{j}\left(p_{\text{M}},{\overset{˙}{p}}_{\text{M}},{\overset{¨}{p}}_{\text{M}},\vec{\nabla_{M}}p_{\text{M}},\vec{\nabla_{M}}{\overset{˙}{p}}_{\text{M}},\vec{\nabla_{M}}{\overset{¨}{p}}_{\text{M}},\eta_{j},{\overset{˙}{\eta }}_{j},{\overset{¨}{\eta }}_{j}\right)=0,\;j=1,...,\text{ɴ}$$

that are also to be obtained from the micro-scale. The macro-scale initial boundary value problem is described by equations (2.1)–(2.3) subjected to initial and boundary conditions. The boundary conditions are then given in terms of pressure (Dirichlet) or gradient of pressure, i.e. velocity (Neumann) boundary conditions, whereas the initial conditions are given for the pressure field, the enrichment variables and their first-time derivatives. In the case of a homogeneous fluid, the conventional representation of the acoustic wave equation in terms of acoustic pressure is achieved after substituting the relations (2.3) and (2.2) into (2.1).

Therefore, the proposed homogenization approach provides an effective fluid behaviour in rigid channels. The core of the proposed framework is to provide a systematic approach to determine the macro-scale constitutive response (2.2), together with the evolution equation (2.3), from the unit cell analysis. It will be achieved through the definition of a scale coupling and homogenization procedure established in §3.

## 3. Micro-scale unit cell problem

### (a) Governing equations

Consider an acoustic metamaterial unit cell where a fluid is constrained to move through a rigid solid structure, as depicted in figure 1*c*. The acoustic wave equation governs the fluid dynamics linearized around a thermodynamic equilibrium state, assuming the fluid is an ideal gas with a quiescent background,


(3.1) $$-\overset{¨}{\epsilon }\left(\vec{x},t\right)+\vec{\nabla }\cdot \overset{¨}{\vec{U}}\left(\vec{x},t\right)=0\;\text{in}\;\Omega_{f}\times \left[t_{1},t_{2}\right],$$

where $\overset{˙}{\epsilon }$ is the volumetric strain rate and $\overset{¨}{\vec{U}}$ the fluid acceleration at the microscopic unit cell scale. These quantities are related to the primary micro-scale unknown field $p\left(\vec{x},t\right)$ , i.e. the fluid pressure disturbance around the background equilibrium (see Landau & Lifshitz [42]), through the constitutive relations of a compressible inviscid fluid


(3.2) $$\begin{array}{c} \overset{˙}{\epsilon }\left(\vec{x},t\right)=-\frac{1}{K_{\text{f}}}\overset{˙}{p}\left(\vec{x},t\right)\;\text{and}\;\overset{¨}{\vec{U}}\left(\vec{x},t\right)=-\frac{1}{\rho_{\text{f}}}\vec{\nabla }p\left(\vec{x},t\right) \end{array}$$

where $\rho_{\text{f}}$ and $K_{\text{f}}$ are the fluid mass density and bulk modulus at the background equilibrium state, respectively. The first equation of (3.2) is the combination of the linearized continuity equation with the linearized state equation, and the second is the linearized balance of linear momentum. Substitution of (3.2) into (3.1) leads to the conventional form of the acoustic wave equation. The dissipation due to viscous effects and heat conduction will be disregarded here. As pointed out in [43,44], the inviscid response gives the leading-order behaviour of the fluid’s effective motion in porous materials for frequencies higher than a minimal frequency $f_{\min}=4\nu_{\text{f}}\;/\left(\pi d^{2}\right)$ , where $\nu_{\text{f}}$ is the kinematic viscosity of the fluid and d the channel’s characteristic width. The sound hard boundary condition is imposed at the interface between the fluid and rigid solid domains as


(3.3) $$\overset{¨}{\vec{U}}\left(\vec{x},t\right)\cdot {\vec{n}}_{\text{fs}}=0\;\text{for}\;\left(\vec{x},t\right)\in \Gamma_{\text{fs}}\times \left[t_{1},t_{2}\right]$$

where ${\vec{n}}_{fs}$ is the normal to the interface. Finally, the resulting partial differential equation, (3.1), with (3.2) and (3.3), is complemented by boundary and initial conditions that are to be derived from the scale coupling relations discussed in §3c.

### (b) The discretized micro-scale problem projected on a reduced space

For the unit cell, a standard finite element discretization scheme is used for the governing equation (3.1), defining the micro-scale problem with so-far-undefined boundary conditions. The procedure leads to the system of second-order ordinary differential equations written in matrix format as


(3.4) $$\underline{Q}\;\underset{∼}{\ddot{p}}\;+\;\underline{H}\;\underset{∼}{p}\;=-{\underset{∼}{q}}^{\text{ ext}}$$

where $\underline{Q}$ is the matrix related to the volumetric strain energy and $\underline{H}$ the matrix related to the kinetic energy. On the right-hand side, ${\underset{∼}{q}}^{\text{ext}}$ is the external acceleration flux imposed at the unit cell boundary, i.e. the acoustic load. The system (3.4) is symmetric. When a time-harmonic response is considered, it has real positive eigenvalues since no dissipative phenomena are included in the micro-scale constitutive models (3.2). The finite element nodes may be subdivided into the nodes at the boundary, where the essential boundary conditions are prescribed, denoted by ‘ ʙ ’, and the remaining ‘independent’ nodes that are internal to the unit cell, denoted by ‘ ɪ ’, resulting in the partitioned form of equation (3.4):


(3.5) $$\begin{array}{c} \left[\begin{array}{c} {\underline{Q}}_{\text{ʙ}\text{ʙ}}\;{\underline{Q}}_{\text{ʙ}\text{ɪ}}\\ {\underline{Q}}_{\text{ɪ}\text{ʙ}}\;{\underline{Q}}_{\text{ɪ}\text{ɪ}} \end{array}\right]\left[\begin{array}{c} {\underset{∼}{\overset{¨}{p}}}_{\text{ʙ}}\\ {\underset{∼}{\overset{¨}{p}}}_{\text{ɪ}} \end{array}\right]+\left[\begin{array}{c} {\underline{H}}_{\text{ʙ}\text{ʙ}}\;{\underline{H}}_{\text{ʙ}\text{ɪ}}\\ {\underline{H}}_{\text{ɪ}\text{ʙ}}\;{\underline{H}}_{\text{ɪ}\text{ɪ}} \end{array}\right]\left[\begin{array}{c} {\underset{∼}{p}}_{\text{ʙ}}\\ {\underset{∼}{p}}_{\text{ɪ}} \end{array}\right]=\left[\begin{array}{c} -{\underset{∼}{q}}_{\text{ʙ}}^{\text{ext}}\\ {\underset{∼}{0}}_{\text{ɪ}} \end{array}\right]. \end{array}$$

Next, the goal is to express the internal degrees of freedom ${\underset{∼}{p}}_{\text{ɪ}}$ in terms of a reduced space.

For the considered wave problem, two distinct and approximately superimposable solutions can be discerned for constructing a representative reduced basis. The first solution assumes a steady-state response subjected to arbitrary Dirichlet boundary conditions on the ʙ boundary nodes:


(3.6) $$\vec{\nabla }\cdot \left(\frac{1}{\rho_{f}}\vec{\nabla }p\right)=0,\;\text{in}\;\Omega_{\text{f}}\times t.$$

The solution to this problem is used to construct the so-called long wavelength basis, i.e. the unit cell response at the limit of a long wavelength propagation, enabling the elimination of the internal degrees of freedom ${\underset{∼}{p}}_{\text{ɪ}}$ . As a result, the solution of the transient unit cell problem (3.5) projected onto the long wavelength basis implies an instantaneous response of the internal pressure nodes ${\underset{∼}{p}}_{\text{ɪ}}$ according to the steady-state condition computed by a given $t\in \left[t_{1},t_{2}\right]$ .

The second solution is the eigenvalue problem


(3.7) $$-\frac{\omega^{2}}{K_{f}}p+\vec{\nabla }\cdot \left(\frac{1}{\rho_{f}}\vec{\nabla }p\right)=0\;\text{in}\;\Omega_{f},$$

where ω is the eigenfrequency in (rad s ${,}^{-1}$ ) of the unit cell subjected to zero Dirichlet boundary conditions. It gives the internal dynamics when the unit cell’s outer boundary is close to a steady state relative to the internal domain, such as the case considered in this article. This condition was introduced in the context of computational homogenization of solid metamaterials [36,39,45–50] and is called the relaxed separation of scales. The solution of the eigenvalue problem (3.7) provides the spectral basis, which using a modal expansion allows reducing the internal degrees of freedom ${\underset{∼}{p}}_{\text{ɪ}}$ to the ɴ lowest relevant eigenmode amplitudes ${\underset{∼}{\eta }}_{\text{ɴ}}$ , with typically ɴ≪ɪ . Indeed, the aim here is to capture the local resonance behaviour using a small spectral basis generated under zero Dirichlet boundary conditions. Sridhar *et al*. [36] demonstrated for a solid metamaterial case that the zero Dirichlet boundary condition leads to a suitable basis. Here, it will be shown that it is equally appropriate as a boundary condition for a fluid metamaterial described in the pressure formulation. Based on the above considerations, the solution to the problem of a fluid unit cell that has localized dynamics (subwavelength resonances) can be represented as the superposition of the long wavelength and local resonance solutions:


(3.8) $$\begin{array}{ccc} & \begin{array}{cccc} & \underset{∼}{p} & \approx {\underset{∼}{p}}^{\text{(long wavelength)}}+{\underset{∼}{p}}^{\text{(local resonance)}}=\left[\begin{array}{c} {\underset{∼}{p}}_{\text{ʙ}}\\ {\underset{∼}{p}}_{\text{ɪ}}^{\text{(lw)}} \end{array}\right]+\left[\begin{array}{c} {\underset{∼}{0}}_{\text{ʙ}}\\ {\underset{∼}{p}}_{\text{ɪ}}^{\text{(lr)}} \end{array}\right]=\left[\begin{array}{c} {\underset{∼}{p}}_{\text{ʙ}}\\ {\underset{∼}{p}}_{\text{ɪ}}^{\text{(lw)}}+{\underset{∼}{p}}_{\text{ɪ}}^{\text{(lr)}} \end{array}\right]. & \end{array} & \end{array}$$

The discrete form of the long wavelength limit equation (3.6) is obtained by disregarding the transient term in equation (3.5). From there, ${\underset{∼}{p}}_{\text{ɪ}}^{\text{(lw)}}$ can be expressed in terms of the long wavelength basis ${\underline{\Sigma }}_{\text{ɪ}\text{ʙ}}$ as


(3.9) $${\underset{∼}{p}}_{\text{ɪ}}^{\text{(lw)}}={\underline{\Sigma }}_{\text{ɪ}\text{ʙ}}\;{\underset{∼}{p}}_{\text{ʙ}}\;\text{with}\;{\underline{\Sigma }}_{\text{ɪ}\text{ʙ}}=-{\underline{H}}_{\text{ɪ}\text{ɪ}}^{-1}{\underline{H}}_{\text{ɪ}\text{ʙ}}.$$

The discretized time-harmonic problem (3.7) is given by


(3.10) $$\;\left[-\omega_{j}^{2}\;\;{\underline{Q}}_{\text{ɪ}\text{ɪ}}\;+\;{\underline{H}}_{\text{ɪ}\text{ɪ}}\right]\;{\underset{∼}{\varphi }}_{\text{I}}^{\left(j\right)}\;=\;{\underset{∼}{0}}_{\text{I}}$$

with ${\underset{∼}{\varphi }}_{\text{I}}^{\left(j\right)}$ the j th eigenmode, where j=1,…,ɪ . Next, a selection of ɴ lowest localized resonant modes will be made, with the truncation ɴ≪ɪ . The selected modes are collected in the matrix ${\underline{\Phi }}_{\text{ɪ}\text{ɴ}}$ referred to as the ‘local resonance’ basis. Thus, the internal nodal pressure variables in the local resonance solution are approximated using the truncated spectral basis


(3.11) $${\underset{∼}{p}}_{\text{ɪ}}^{\text{(lr)}}\approx {\underline{\Phi }}_{\text{ɪɴ}}\;{\underset{∼}{\eta }}_{\text{ɴ}}$$

with ${\underset{∼}{\eta }}_{\text{ɴ}}$ the column of modal amplitudes. The matrix ${\underline{\Phi }}_{\text{ɪ}\text{ɴ}}$ is normalized with respect to the volumetric strain matrix, i.e. satisfying


(3.12) $${\underline{\Phi }}_{\text{ɪɴ}}^{\text{T}}\;{\underline{Q}}_{\text{ɪɪ}}\;{\underline{\Phi }}_{\text{ɪɴ}}={\underline{I}}_{\text{ɴɴ}}^{\text{(lr)}}\;\text{and hence}\;{\underline{\Lambda }}_{\text{ɴɴ}}^{\text{(lr)}}={\underline{\Phi }}_{\text{ɪɴ}}^{\text{T}}\;{\underline{H}}_{\text{ɪɪ}}\;{\underline{\Phi }}_{\text{ɪɴ}}$$

with ${\underline{I}}_{\text{ɴɴ}}^{\text{(lr)}}$ the identity matrix of the appropriate dimension. As a direct consequence, the second equation of (3.12) is the kinetic energy matrix expressed in the modal basis. The diagonal matrix contains the truncated eigenvalues $\omega_{j}^{2}$ , where j=1,…,ɴ . Collecting (3.9) and (3.11) into (3.8), the transformation $\underline{A}$ resulting from the superposition of the solutions projected on the reduced bases is


(3.13) $$\begin{array}{c} \left[\begin{array}{c} {\underset{∼}{p}}_{\text{ʙ}}\\ {\underset{∼}{p}}_{\text{ɪ}} \end{array}\right]=\left[\begin{array}{c} {\underline{0}}_{\text{ʙɴ}}\;{\underline{I}}_{\text{ʙʙ}}\\ {\underline{\Phi }}_{\text{ɪɴ}}\;{\underline{\Sigma }}_{\text{ɪʙ}} \end{array}\right]\left[\begin{array}{c} {\underset{∼}{\eta }}_{\text{ɴ}}\\ {\underset{∼}{p}}_{\text{ʙ}} \end{array}\right]\equiv \underline{A}\left[\begin{array}{c} {\underset{∼}{\eta }}_{\text{ɴ}}\\ {\underset{∼}{p}}_{\text{ʙ}} \end{array}\right], \end{array}$$

where $\underline{0}$ and $\underline{I}$ are the zero and identity matrices, respectively. Equation (3.5) can then be projected on the reduced basis


(3.14) $$\begin{array}{c} \left({\underline{A}}^{T}\left[\begin{array}{c} {\underline{Q}}_{\text{ʙ}\text{ʙ}}\;{\underline{Q}}_{\text{ʙ}\text{ɪ}}\\ {\underline{Q}}_{\text{ɪ}\text{ʙ}}\;{\underline{Q}}_{\text{ɪ}\text{ɪ}} \end{array}\right]\underline{A}\right)\left[\begin{array}{c} {\underset{∼}{\overset{¨}{\eta }}}_{\text{ɴ}}\\ {\overset{¨}{\underset{∼}{p}}}_{\text{ʙ}} \end{array}\right]+\left({\underline{A}}^{T}\left[\begin{array}{c} {\underline{H}}_{\text{ʙ}\text{ʙ}}\;{\underline{H}}_{\text{ʙ}\text{ɪ}}\\ {\underline{H}}_{\text{ɪ}\text{ʙ}}\;{\underline{H}}_{\text{ɪ}\text{ɪ}} \end{array}\right]\underline{A}\right)\left[\begin{array}{c} {\underset{∼}{\eta }}_{\text{ɴ}}\\ {\underset{∼}{p}}_{\text{ʙ}} \end{array}\right]={\underline{A}}^{T}\left[\begin{array}{c} -{\underset{∼}{q}}_{\text{ʙ}}^{\text{ext}}\\ {\underset{∼}{0}}_{\text{ɪ}} \end{array}\right] \end{array}$$

leading to


(3.15) $${\underline{I}}_{\text{ɴɴ}}^{\text{(lr)}}\;{\ddot{\underset{∼}{\eta }}}_{\text{ɴ}}+{\underline{\Lambda }}_{\text{ɴɴ}}^{\text{(lr)}}\;{\underset{∼}{\eta }}_{\text{ɴ}}\;\;=\;\;-{\underline{m}}_{\text{ɴʙ}}^{\text{(c)}}\;{\ddot{\underset{∼}{p}}}_{\text{ʙ}}\;$$

(3.16) $${\underline{Q}}_{\text{ʙ}\text{ʙ}}^{\text{(lw)}}\;{\underset{∼}{\overset{¨}{p}}}_{\text{ʙ}}\;+\;{\underline{H}}_{\text{ʙ}\text{ʙ}}^{\text{(lw)}}\;{\underset{∼}{p}}_{\text{ʙ}}\;+\;{\underline{m}}_{\text{ʙɴ}}^{\text{(c)}}\;{\underset{∼}{\overset{¨}{\eta }}}_{\text{ɴ}}\;=\;-{\underset{∼}{q}}_{\text{ʙ}}^{\text{ext}},$$

where


(3.17) $$\begin{array}{l} {\underline{Q}}_{\text{ʙʙ}}^{\text{(lw)}}={\underline{Q}}_{\text{ʙʙ}}+{\underline{Q}}_{\text{ʙɪ}}\;{\underline{\Sigma }}_{\text{ɪʙ}}+{\underline{\Sigma }}_{\text{ɪʙ}}^{T}\;{\underline{Q}}_{\text{ɪʙ}}+{\underline{\Sigma }}_{\text{ɪʙ}}^{T}\;{\underline{Q}}_{\text{ɪɪ}}\;{\underline{\Sigma }}_{\text{ɪʙ}}\\ {\underline{H}}_{\text{ʙʙ}}^{\text{(lw)}}={\underline{H}}_{\text{ʙʙ}}+{\underline{H}}_{\text{ʙɪ}}\;{\underline{\Sigma }}_{\text{ɪʙ}}+{\underline{\Sigma }}_{\text{ɪʙ}}^{T}\;{\underline{H}}_{\text{ɪʙ}}+{\underline{\Sigma }}_{\text{ɪʙ}}^{T}\;{\underline{H}}_{\text{ɪɪ}}\;{\underline{\Sigma }}_{\text{ɪʙ}} \end{array}\;\;\text{and}\;\;\begin{array}{l} {\underline{m}}_{\text{ɴʙ}}^{\text{(c)}}={\underline{\Phi }}_{\text{ɪɴ}}^{T}\;\;{\underline{Q}}_{\text{ɪʙ}}+{\underline{\Phi }}_{\text{ɪɴ}}^{T}\;\;{\underline{Q}}_{\text{ɪɪ}}\;\;{\underline{\Sigma }}_{\text{ɪʙ}}\\ {\underline{m}}_{\text{ʙɴ}}^{\text{(c)}}={\underline{Q}}_{\text{ʙɪ}}\;\;{\underline{\Phi }}_{\text{ɪɴ}}+{\underline{\Sigma }}_{\text{ɪʙ}}^{T}\;\;{\underline{Q}}_{\text{ɪɪ}}\;\;{\underline{\Phi }}_{\text{ɪɴ}}. \end{array}$$

The matrices ${\underline{Q}}_{\text{ʙ}\text{ʙ}}^{\text{(lw)}}$ , ${\underline{H}}_{\text{ʙ}\text{ʙ}}^{\text{(lw)}}$ , ${\underline{m}}_{\text{ʙɴ}}^{\text{(c)}}$ and ${\underline{m}}_{\text{ɴʙ}}^{\text{(c)}}$ are the finite element submatrices of (3.5) projected on the reduced basis. Since the involved matrices are symmetric, it holds that ${\underline{m}}_{\text{ʙɴ}}^{\text{(c)}}={\left({\underline{m}}_{\text{ɴʙ}}^{\text{(c)}}\right)}^{\text{T}}$ . Note that the coupling between the local resonance dynamics (3.15) and the long wavelength dynamics (3.16), given by the matrices ${\underline{m}}_{\text{ɴʙ}}^{\text{(c)}}$ and ${\underline{m}}_{\text{ʙɴ}}^{\text{(c)}}$ , is purely driven by the inertial forces as these matrices depend only on the matrix $\underline{Q}$ and the reduced basis.

### (c) The scale coupling

First-order spatial computational homogenization [37] expands the microscopic field variables in terms of the macroscopic fields and their gradients. For the acoustics problem considered here, the micro-scale pressure field $p\left(\vec{x},t\right)$ is represented as


(3.18) $$p=p_{\text{M}}+(\vec{x}-\vec{x_{R}})\cdot (\vec{\nabla_{\text{M}}}p_{\text{M}})+w_{p}\;\;\;\;\text{for}\;\;\vec{x}\;\in \;\Omega_{f}.$$

The micro-fluctuation field $w_{p}\left(\vec{x},t\right)$ is added to accommodate the difference between the macro-scale field and the local variations that may develop within the unit cell due to the presence of inhomogeneities; $\vec{x_{R}}$ is a reference position vector, here chosen to be placed at the centre of the unit cell.

To couple the scales, in computational homogenization methods, an equivalence between the generalized power at the two scales is established, i.e. the so-called Hill–Mandel condition. To this end, the variational formulation of the macroscopic problem is extracted from equation (2.1). The integral functional is written as


(3.19) $$\int_{\Omega_{\text{M}}}\left\{-{\overset{¨}{\epsilon }}_{\text{M}}+\vec{\nabla_{\text{M}}}\cdot {\overset{¨}{\vec{U}}}_{\text{M}}\right\}\delta p_{\text{M}}{dV}_{\text{M}}=0,\;\forall \delta p_{\text{M}}.$$

Using the divergence theorem, (3.19) can be rewritten as


(3.20) $$\int_{\Omega_{\text{M}}}\left\{-{\ddot{\epsilon }}_{\text{M}}\delta p_{\text{M}}-{\ddot{\vec{U}}}_{\text{M}}\cdot \delta \vec{\nabla_{\text{M}}}p_{\text{M}}\right\}{dV}_{\text{M}}=\int_{\partial \Omega_{\text{M}}}-({\ddot{\vec{U}}}_{\text{M}}\cdot \vec{n_{\text{M}}})\delta p_{\text{M}}{dA}_{\text{M}},\;\;\;\;\;\forall \delta p_{\text{M}},$$

where $\vec{n_{\text{M}}}$ is the outward normal to the external boundary of the macroscopic domain $\Omega_{\text{M}}$ . In the left-hand side of equation (3.20), the first term is related to the elastic power rate, and the second term gives the kinetic power rate. The right-hand side of equation (3.20) describes the external virtual power rate applied to the macroscopic surface $\partial \Omega_{\text{M}}$ . A similar procedure is performed for the micro-scale equation (3.1) on the unit cell domain $\Omega_{f}$ . Then, the integral function of the micro-scale problem takes the same form as equation (3.20) with the additional $\Gamma_{f\;s}$ -internal interface term, on the left-hand side, which vanishes due to the interface condition (3.3), leading to


(3.21) $$\int_{\Omega_{f}}\left\{-\ddot{\epsilon }\delta p-\ddot{\vec{U}}\cdot \delta \vec{\nabla }p\right\}dV+\int_{\Gamma_{fs}}\overset{=0,\;b.c.\;(3.3)}{\overbrace{\left(\ddot{\vec{U}}\cdot {\vec{n}}_{fs}\right)}}\delta p\;dA=\int_{\partial \Omega_{f}}-(\ddot{\vec{U}}\cdot \vec{n_{f}})\delta p\;dA,\;\;\forall \delta p,$$

where $\vec{n_{f}}$ is the outward normal to the outer boundary of the fluid unit cell domain $\Omega_{f}$ .

Given the variational forms at macro- (3.20) and micro-scales (3.21), the link between the scales is recovered by equating the local macro-scale virtual power to the unit cell volume averaged power. Hence, the extended Hill–Mandel principle becomes


(3.22) $$\begin{array}{l} \;-{\ddot{\epsilon }}_{\text{M}}\delta p_{\text{M}}-{\ddot{\vec{U}}}_{\text{M}}\cdot \vec{\nabla_{\text{M}}}\delta p_{\text{M}}=\frac{1}{V_{f}}\int_{\Omega_{f}}\left\{-\ddot{\epsilon }\delta p-\ddot{\vec{U}}\cdot \vec{\nabla }\delta p\right\}dV\;\forall (\delta p_{\text{M}},\delta p). \end{array}$$

The volume of the fluid domain $V_{f}$ is related to the total unit cell volume V by the porosity factor $\phi =V_{f}/V$ . Using this definition and substituting (3.21) for the right-hand side of (3.22) gives


(3.23) $$\begin{array}{l} \;-\phi {\ddot{\epsilon }}_{\text{M}}\delta p_{\text{M}}-\phi {\ddot{\vec{U}}}_{\text{M}}\cdot \delta \vec{\nabla_{\text{M}}}p_{\text{M}}=\frac{1}{V}\int_{\partial \Omega_{f}}-(\ddot{\vec{U}}\cdot \vec{n_{f}})\delta p\;\;dA,\;\;\forall (\delta p_{\text{M}},\delta p). \end{array}$$

Substituting the macro-to-micro relation (3.18) into (3.23) gives


(3.24) $$\begin{array}{l} \;-\phi {\ddot{\epsilon }}_{\text{M}}\delta p_{\text{M}}-\phi {\ddot{\vec{U}}}_{\text{M}}\cdot \delta \vec{\nabla_{\text{M}}}p_{\text{M}}=\frac{1}{V}\int_{\partial \Omega_{f}}(-\ddot{\vec{U}}\cdot \vec{n_{f}})\left(\delta p_{\text{M}}+(\vec{x}-\vec{x_{R}})\cdot \delta (\vec{\nabla_{\text{M}}}p_{\text{M}})+\delta w_{p}\right)dA,\;\forall (\delta p_{\text{M}},\delta w_{p}) \end{array}$$

As required in any computational homogenization approach, the virtual power of the micro-fluctuations on the boundary is forced to vanish by using appropriate boundary conditions, i.e.


(3.25) $$\begin{array}{r} \int_{\partial \Omega_{f}}-(\ddot{\vec{U}}\cdot \vec{n_{f}})\;\delta w_{p}\;dA=0. \end{array}$$

For instance, this is ensured by *zero uniform* boundary condition on the micro-fluctuation, referred to as ‘*null* boundary condition’ by Blanco *et al*. [51]


(3.26) $$\begin{array}{r} w_{p}(\vec{x})=0,\;\forall \vec{x}\in \partial \Omega_{f}. \end{array}$$

Since equation (3.24) must hold for all admissible macro- and micro-fields, the macro-constitutive relations are obtained from the respective boundary integral relations, which can also be expressed as volume integrals by using the divergence theorem and the balance equation (3.1)


(3.27) $$\begin{array}{l} \phi {\ddot{\epsilon }}_{\text{M}}=\frac{1}{V}\int_{\partial \Omega_{f}}(\ddot{\vec{U}}\cdot \vec{n_{f}})\;\;\;dA\\ \phi {\ddot{\vec{U}}}_{\text{M}}=\frac{1}{V}\int_{\partial \Omega_{f}}(\ddot{\vec{U}}\cdot \vec{n_{f}})(\vec{x}-\vec{x_{R}})\;\;\;dA, \end{array}\;\;\text{or}\;\;\begin{array}{l} \phi {\ddot{\epsilon }}_{\text{M}}=\frac{1}{V}\int_{\Omega_{f}}\ddot{\epsilon }\;\;\;dV\;\;\\ \phi {\ddot{\vec{U}}}_{\text{M}}=\frac{1}{V}\int_{\Omega_{f}}\left\{\ddot{\vec{U}}+\ddot{\epsilon }\;(\vec{x}-\vec{x_{R}})\right\}\;dV. \end{array}$$

For solid locally resonant metamaterials, the incorporation of the micro-inertia contribution into the macro-scale flux (stress) was introduced by Pham *et al*.[39]. Here, an analogous expression is found for the macro-scale flux (acceleration) ${\overset{¨}{\vec{U}}}_{\text{M}}$ which depends not only on the micro-scale field $\overset{¨}{\vec{U}}$ but also on the micro-inertia term $\overset{¨}{\epsilon }\left(\vec{x}-\vec{x_{R}}\right)$ , see the right-hand volume integral of (3.27). This resulting expression is the transient version of the time-harmonic homogenization developed by Gao *et al*. [52] for a deformable porous solid saturated by a fluid.

Finally, it is instructive to establish the scale coupling relations between the macro- and micro-scale pressure and gradient of pressure fields. The averaging relation for the pressure gradient can be found by applying the micro-scale gradient on (3.18) and integrating over the unit cell *volume* $\Omega_{f}$ , followed by using the divergence theorem on the term involving micro-fluctuations. With regard to ansatz (3.26), the latter term vanishes, and the macro-to-micro constraint for the pressure gradient is derived


$$\begin{array}{c} \vec{\nabla_{M}}p_{M}=\frac{1}{V_{f}}\int_{\Omega_{f}}\vec{\nabla }p\;dV. \end{array}$$

Next, the averaging relation for the pressure can be found by integrating equation (3.18) over the unit cell *boundary* and taking $\vec{x_{R}}$ in the centroid of the unit cell, with account for equation (3.26), the terms $\int_{\partial \Omega_{f}}\left(\vec{x}-\vec{x_{R}}\right)dA$ and $\int_{\partial \Omega_{f}}w_{p}dA$ vanish. Then, the constraint for the pressure is obtained


$$\begin{array}{c} p_{M}=\frac{1}{A_{f}}\int_{\partial \Omega_{f}}p\;dA, \end{array}$$

where $A_{f}=\int_{\partial \Omega_{f}}dA$ . This macro–micro constraint aligns with the classic asymptotic and computational homogenization of locally resonant materials where the macro-scale field represents the averaged field of the non-resonant domain, e.g. see Boutin *et al*. [40], Pham *et al*. [39] and Sridhar *et al*. [36].

### (d) Upscaling the reduced micro-scale response

Considering the infinitesimal acceleration flux $dq^{\text{ext}}=\ddot{\vec{U}}\cdot \vec{n_{f}}\;dA$ , for the discretized unit cell problem, the scale coupling relations (3.27) can be written as


(3.28) $$\begin{array}{ccc} \phi {\overset{¨}{\epsilon }}_{\text{M}}=\frac{1}{V}\;{\underset{∼}{1}}_{\text{ʙ}}^{T}\;{\underset{∼}{q}}_{\text{ʙ}}^{\text{ext}} & \text{and} & \phi {\ddot{\vec{U}}}_{\text{M}}=\frac{1}{V}\;{\left({\underset{∼}{q}}_{\text{ʙ}}^{\text{ext}}\right)}^{T}\Delta {\underset{∼}{\vec{x}}}_{\text{ʙ}}, \end{array}$$

where ${\underset{∼}{1}}_{\text{ʙ}}$ is a column with scalar unit components. The notation $\Delta {\vec{x}}_{\text{ʙ}}={\vec{x}}_{\text{ʙ}}-\vec{x_{R}}$ is introduced for a position vector on the unit cell boundary ${\vec{x}}_{\text{ʙ}}\in \partial \Omega_{f}$ and, in discrete form, it is written as $\Delta {\underset{∼}{\vec{x}}}_{\text{ʙ}}$ . Taking into account equations (3.18) and (3.26), the pressure at the boundary nodes is expressed as a function of the macroscopic state as


(3.29) $$\begin{array}{c} \begin{array}{c} {\underset{∼}{p}}_{\text{ʙ}}={\underset{∼}{1}}_{\text{ʙ}}\;p_{\text{M}}+\Delta {\underset{∼}{\vec{x}}}_{\text{ʙ}}\cdot \vec{\nabla_{\text{M}}}p_{\text{M}}. \end{array} \end{array}$$

Next, equation (3.29) is inserted into (3.16), and finally, ${\underset{∼}{q}}_{\text{ʙ}}^{\text{ext}}$ from (3.16) is substituted in (3.28), resulting in closed-form expressions of the constitutive relations for the macroscopic fluid volumetric strain $\epsilon_{\text{M}}$ , and macroscopic fluid displacement ${\vec{U}}_{\text{M}}$ as functions of the macroscopic state $\left(p_{\text{M}},\vec{\nabla_{\text{M}}}p_{\text{M}}\right)$ and the modal amplitudes ${\underset{∼}{\eta }}_{\text{ɴ}}$


(3.30) $$\begin{array}{ccc} & \begin{array}{c} \phi {\overset{¨}{\epsilon }}_{\text{M}}=C_{\epsilon }\;{\overset{¨}{p}}_{\text{M}}+\vec{D_{\epsilon }}\cdot \vec{\nabla_{\text{M}}}{\overset{¨}{p}}_{\text{M}}+G_{\epsilon }\;p_{\text{M}}+\vec{H_{\epsilon }}\cdot \vec{\nabla_{\text{M}}}p_{\text{M}}+\sum_{j=1}^{\text{ɴ}}c_{j}\;\overset{¨}{\eta_{j}}\\ \phi {\overset{¨}{\vec{U}}}_{\text{M}}=\vec{C_{U}}\;{\overset{¨}{p}}_{\text{M}}+D_{U}\cdot \vec{\nabla_{\text{M}}}{\overset{¨}{p}}_{\text{M}}+\vec{G_{U}}\;p_{\text{M}}+H_{U}\cdot \vec{\nabla_{\text{M}}}p_{\text{M}}+\sum_{j=1}^{\text{ɴ}}\vec{d_{j}}\;\overset{¨}{\eta_{j}} \end{array} & \end{array}$$

where the upper-case homogenized material coefficients $C_{\xi }$ , $D_{\xi }$ , $G_{\xi }$ and $H_{\xi }$ , with subscript ξ={ϵ,U} , correspond to the long wavelength regime, and the lower-case ones $c_{j}$ and $\vec{d_{j}}$ are the homogenized material coefficients of mode j , corresponding to the local resonance regime. Furthermore, combining equation (3.15) with (3.29) yields the evolution equation for the internal dynamic variables ${\underset{\sim }{\eta }}_{\text{N}}$


(3.31) $$\overset{¨}{\eta_{j}}+\;\;\omega_{j}^{2}\eta_{j}\;=\;\;\;\gamma_{j}\;\;{\overset{¨}{p}}_{\text{M}}\;\;\;\;+\;\;\;\vec{\delta_{j}}\cdot \vec{\nabla_{\text{M}}}{\overset{¨}{p}}_{\text{M}}\;,\;\;\text{for eigenmodes}\;\;j=1,2,...,\text{ɴ},$$

where $\gamma_{j}$ and $\vec{\delta_{j}}$ are the homogenized material coefficients of mode j , corresponding to the local resonance regime, where


(3.32) $$\gamma_{j}=Vc_{j},\;\text{and}\;\vec{\delta_{j}}=V\vec{d_{j}},\;\;\text{for eigenmodes }j=1,2,...,\text{ɴ}.$$

The detailed derivations leading to constitutive relations (3.30) and the evolution equation (3.31), as well as the final expressions of the homogenized coefficients, are given in appendix A, where it is also shown that the coefficients $G_{\epsilon }$ , $\vec{H_{\epsilon }}$ and $\vec{G_{U}}$ are identically zero. The long wavelength limits material properties $C_{\epsilon }$ and ${𝐇}_{U}$ are the conventional coefficients in the constitutive models in homogeneous (non-porous) fluid acoustics, and they are typically non-negligible, as expected since the homogeneous fluid is a limit case for ϕ→1 . The effective macro-scale bulk modulus and mass density tensor are, respectively, $K_{\text{M}}=-C_{\epsilon }^{-1}$ and ${𝝆}_{\text{M}}=-{𝐇}_{U}^{-1}$ . Coefficients ${𝐃}_{U}$ and $\vec{D_{\epsilon }}=\vec{C_{U}}$ are similar to the so-called Willis coefficients [24] and naturally emerge from the spatial unit cell averaging. The latter may or may not vanish depending on the unit cell symmetries and the specific choice of $\vec{x_{R}}$ , even in the long wavelength limit, which agrees with the findings of Willis [53]. The local resonance coefficients $c_{j}$ and $\vec{d_{j}}$ depend, in general, on the nature of the occurring subwavelength resonance. For instance, a monopolar resonance induces a negative bulk modulus, leading to a large corresponding coefficient $c_{j}$ . On the other hand, if a dipolar resonance is present, it induces a negative effective mass, resulting in a large coefficient $\vec{d_{j}}$ .

**Figure 2.**
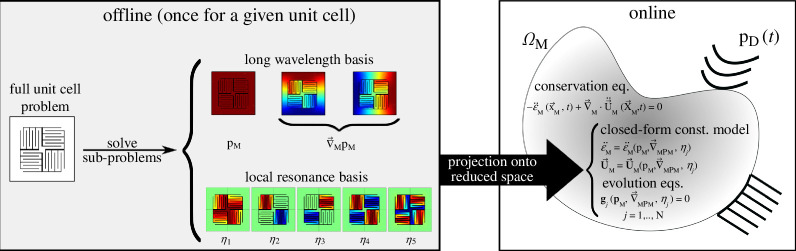
The summary of the reduced-order computational homogenization steps for constructing an equivalent macro-scale model from the selection of a unit cell.

To summarize, at the macro level, an enriched homogenized continuum has emerged. The internal degrees of freedom ${\underset{∼}{p}}_{\text{ɪ}}$ , governed by equation (3.5), were projected onto the reduced space identified in §3b and upscaled as a superposition of the long wavelength and local resonance responses in §3d. Note that this model reduction and projection need to be done only once for a given unit cell, thus justifying the computational costs involved. The resulting formulation emerging at the macro-scale can be denoted as $\left(p_{\text{M}},{\underset{∼}{\eta }}_{\text{ɴ}}\right)$ , with $p_{\text{M}}$ and ${\underset{∼}{\eta }}_{\text{ɴ}}$ being the primary unknown fields. By virtue of the closed-form constitutive relations (3.30), the unit cell no longer has to be consulted, revealing a fundamental difference with respect to the FE ${,}^{2}$ computational homogenization approach. To conclude, the enriched homogenized continuum, depicted in figure 1*b*, is governed by the conservation equation (2.1), the constitutive model (3.30) and the ɴ evolution equation (3.31) for ${\underset{∼}{\eta }}_{\text{ɴ}}$ enrichment variables. The latter can be interpreted to provide the enrichment of the homogenized continuum in a micromorphic sense. Figure 2 summarizes the multiscale procedure to construct the effective macro-scale model.

## 4. Numerical example

**Figure 3.**
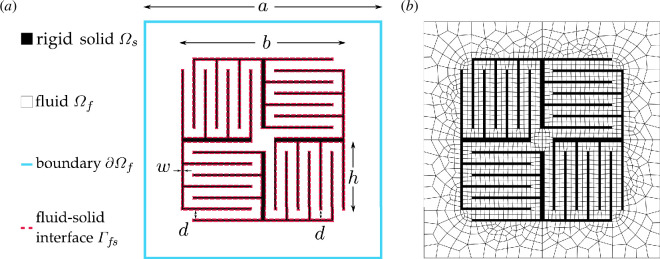
(*a*) Locally resonant labyrinthine metamaterial with a rigid solid structure that coils the wave path. (*b*) Finite element mesh for the fluid domain.

To illustrate the capabilities of the presented framework, an acoustic locally resonant metamaterial is considered, based on the working principles presented by Cheng *et al*. [8]. The unit cell of size a contains a rigid solid labyrinthine-like structure, with size b , immersed in a fluid modelled in two dimensions, as shown in figure 3. The coiled space region triggers the fluid resonance inside the rigid structure in a subwavelength regime relative to the non-coiled region. For the unit cell in figure 3, when an acoustic wave in the non-coiled region travels a distance b/2 , the wave inside the labyrinth travels a distance of approx. 7b/2 through the labyrinth. Effectively, the wave speed is therefore reduced by a factor of 7 .

Air is considered as the background fluid with $\rho_{f}=1.225$ kg m^−^ ${,}^{3}$ and $K_{f}=1.441\times 10^{5}$ Pa at a temperature of $15^{\circ }$ C. The values of the geometric features indicated in figure 3*a* are a=105 , b=72.7 , d=4.05 , h=31.3 and w=1 in mm. As discussed in §3a, given the dynamic viscosity of air $\nu_{f}=1.802\times 10^{-5}\;m^{2}\;s^{-1}$ and the smallest constriction d , the minimum frequency $f_{\text{min}}$ , for which the governing equation holds, is estimated to be 2 Hz. Figure 3*b* shows the two-dimensional finite element mesh used for the unit cell analysis. Quadratic serendipity elements are used with eight pressure nodes per two-dimensional element.

**Figure 4.**
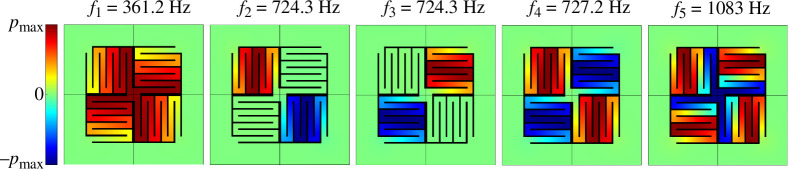
The lowest five eigenmodes constitute the truncated spectral basis with ɴ=5 . The coloured domain is the fluid phase, whereas the rigid solid domain is shown in black.

Figure 4 shows the five lowest (local resonance) modes resulting from the eigenvalue problem (equation (3.7)) with zero Dirichlet boundary conditions. These modes constitute the spectral basis of size ɴ=5 .

Table 1 summarizes the constitutive parameters from (3.30) computed for the considered unit cell. Due to the symmetries of the unit cell, the homogenized fluid density $\rho_{\text{M}}$ and non-classic coefficient $\vec{D_{U}}$ are isotropic, while the Willis-like coefficients $\vec{D_{\epsilon }}$ and $\vec{C_{U}}$ vanish. The presence of the rigid channels forces the fluid to move through a tortuous path, yielding an apparent increase of density, i.e. the homogenized fluid density $\rho_{\text{M}}$ is greater than the background fluid density $\rho_{f}$ . On the other hand, for the homogenized bulk modulus $K_{\text{M}}$ , originating from the long wavelength limit basis, the equality $K_{\text{M}}=K_{f}/\phi$ holds, underlining the consistency of the framework with the analytically derived effective bulk modulus of a fluid with immersed rigid rods in the longwave limit as discussed by Hu *et al*. [30] and Li & Chan [3].

**Table 1.** Homogenized material properties as defined in equation (3.30) computed for the unit cell of figure 3 (see also appendix A).

type	symbol	value	unit
porosity	ϕ	0.8895	[]
long wavelength	$K_{\text{M}}=-C_{\epsilon }^{-1}$	0.1620	[MPa]
long wavelength	$\rho_{\text{M}}=-H_{U}^{-1}$	$3.328\vec{e_{1}}⊗\vec{e_{1}}+3.328\vec{e_{2}}⊗\vec{e_{2}}$	$\left[\mathrm{kg}m^{-3}\right]$
long wavelength	${𝐃}_{U}$	$-\left(0.7319\vec{e_{1}}⊗\vec{e_{1}}+0.7319\vec{e_{2}}⊗\vec{e_{2}}\right)\times 10^{-8}$	$\left[m^{2}Pa^{-1}\right]$
local resonance	$c_{1}$	$\;\;\;\;1.377\times 10^{-2}$	** [] **
local resonance	$c_{5}$	$\;\;\;\;4.772\times 10^{-3}$	** [] **
local resonance	$c_{2}=c_{3}=c_{4}$	0	** [] **
local resonance	$\vec{d_{2}}$	$-\left(0.1966\;\vec{e_{1}}+0.2062\;\vec{e_{2}}\right)\times 10^{-3}$	[m]
local resonance	$\vec{d_{3}}$	$-\left(0.2062\;\vec{e_{1}}-0.1966\;\vec{e_{2}}\right)\times 10^{-3}$	[m]
local resonance	$\vec{d_{1}}=\vec{d_{4}}=\vec{d_{5}}$	$\vec{0}$	[m]
local resonance	${\underset{\sim }{f}}_{\text{modes}}$ (figure 4)	[361.2724.3724.3727.21083]	[Hz]

The dispersion curve of the enriched homogenized continuum can be calculated from the governing equations (2.1), (3.30) and (3.31), by assuming space- and time-harmonicity of the primary field variables $\left(p_{\text{M}},{\underset{∼}{\eta }}_{\text{ɴ}}\right)=\left({\hat{p}}_{\text{M}},{\underset{∼}{\hat{\eta }}}_{\text{ɴ}}\right)e^{i\omega t-i\vec{k}\cdot {\vec{x}}_{\text{M}}}$ . Expressing the sums in equation (3.30) in matrix format for using a compact notation, e.g. $\sum_{j=1}^{\text{ɴ}}\vec{d_{j}}\;{\ddot{\eta }}_{j}={\underset{∼}{\vec{d}}}^{\text{T}}{\ddot{\underset{∼}{\eta }}}_{\text{ɴ}}$ , the following eigenvalue problem is obtained


(4.1) $$\left(\left(-\omega^{2}\right)\left[\begin{array}{ll} C_{\epsilon }+\vec{k}\cdot D_{U}\cdot \vec{k} & \;(\underset{∼}{c}+i\vec{k}\cdot \underset{∼}{\vec{d}}{)}^{T}\\ -\underset{∼}{\gamma }+i\vec{k}\cdot \underset{∼}{\vec{\delta }} & \;{\underline{I}}_{\text{ɴ}\text{ɴ}}^{\text{(lr)}} \end{array}\right]+\left[\begin{array}{ll} \vec{k}\cdot H_{U}\cdot \vec{k} & \;{\underset{∼}{0}}_{\text{ɴ}}^{T}\\ {\underset{∼}{0}}_{\text{ɴ}} & \;{\underline{\Lambda }}_{\text{ɴ}\text{ɴ}}^{\text{(lr)}} \end{array}\right]\right)\left(\begin{array}{l} {\hat{p}}_{\text{M}}\\ {\underset{∼}{\hat{\eta }}}_{\text{ɴ}} \end{array}\right)=\left(\begin{array}{l} 0\\ {\underset{∼}{0}}_{\text{ɴ}} \end{array}\right).$$

**Figure 5.**
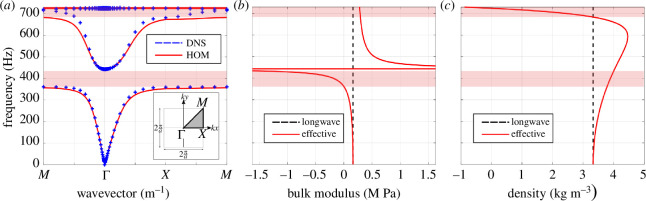
(*a*) Dispersion curves obtained through the computational homogenization approach plotted as a solid red line (**—**) versus the dispersion curves computed using the direct numerical simulation Bloch analysis (DNS) plotted as blue crosses (**+**). (*b*) Effective bulk modulus and (*c*) effective mass density, component $\vec{e_{1}}⊗\vec{e_{1}}$ (equals to component $\vec{e_{2}}⊗\vec{e_{2}}$ ), obtained via the computational homogenization approach given by the expressions defined in equation (4.2).

The above system is Hermitian as follows from the relation between the off-diagonal terms given in (3.32), which is noticeable once the first line is pre-multiplied by the scalar constant −V (the unit cell volume). For a given real-valued wavevector $\vec{k}$ spanning the contour of the irreducible Brillouin zone ( M−Γ−X−M ), the corresponding (real) eigenvalue $\omega^{2}$ can be obtained by solving (4.1), which is plotted as a solid red line (**—**) in figure 5*a*. To validate this result, a direct numerical simulation of the fully resolved unit cell is conducted by applying Bloch–Floquet periodic boundary conditions, providing the reference solution for the dispersion spectrum, shown as blue crosses (**+**) in figure 5*a*. The Bloch analysis in this direct numerical simulation involves approx. ʙ+ɪ≈6000 degrees of freedom. In contrast, the online stage of the homogenization approach contains only 6 degrees of freedom, corresponding to the size of the matrices in (4.1), which is 1+ɴ=6 . It can be observed that the dispersion branch is very well predicted by the homogenized model. A discrepancy is observed in the second branch for frequencies above approx. 550 Hz when the separation of scales is gradually violated.

Frequency-dependent effective material properties can be obtained by assuming time-harmonicity and eliminating the enrichment variables $\eta_{j}$ in the constitutive model by inserting equation (3.31) into (3.30). Thus, the frequency domain effective homogenized constitutive relations read as


(4.2) $$\phi \epsilon_{M}=\overset{-{\left(K_{M}^{\text{(eff)}}(\omega )\right)}^{-1}\equiv }{\overbrace{\left[C_{\epsilon }+\sum_{j=1}^{N}\frac{-\omega^{2}}{-\omega^{2}+\omega_{j}^{2}}\;c_{j}\gamma_{j}\right]}}p_{M}+\overset{{\vec{S_{M}}}^{\text{(eff)}}(\omega )\equiv }{\overbrace{\left[{\vec{D}}_{\epsilon }+\sum_{j=1}^{N}\frac{-\omega^{2}}{-\omega^{2}+\omega_{j}^{2}}\;c_{j}\vec{\delta_{j}}\right]}}\cdot \vec{\nabla_{M}}p_{M}\;\phi {\ddot{\vec{U}}}_{M}=\overset{={\vec{S_{M}}}^{\text{(eff)}}(\omega ),\;due\;to\;(3.32),\;(A3),\;and\;(A4)}{\overbrace{\left[{\vec{C}}_{U}+\sum_{j=1}^{N}\frac{-\omega^{2}}{-\omega^{2}+\omega_{j}^{2}}\;\vec{d_{j}}\gamma_{j}\right]}}{\ddot{p}}_{M}+\overset{-{\left(\rho_{M}^{\left(eff\right)}(\omega )\right)}^{-1}\equiv }{\overbrace{\left[-\omega^{2}D_{U}+H_{U}+\sum_{j=1}^{N}\frac{-\omega^{2}}{-\omega^{2}+\omega_{j}^{2}}\vec{d_{j}}⊗\vec{\delta_{j}}\right]}}\cdot \vec{\nabla_{M}}p_{M}$$

where $K_{\text{M}}^{\text{(eff)}}\left(\omega \right)$ and $\rho_{\text{M}}^{\text{(eff)}}\left(\omega \right)$ are the homogenized effective bulk modulus and effective density, respectively. These are shown in figure 5*b*,*c* as a solid red line (**—**), whereas the long wavelength limit values, i.e. for ω→0 , are shown as dashed black lines (** −− **). The first bandgap (red-shaded range around 400 Hz) is related to the negative effective bulk modulus, while the second bandgap exhibited by the homogenized model (red-shaded range around 700 Hz) relates to a negative effective density. The effective Willis-like coupling coefficient ${\vec{S_{\text{M}}}}^{\text{(eff)}}\left(\omega \right)$ is approximately zero even in the vicinity of the resonances for the considered unit cell and is therefore not shown here. Interestingly, it is observed that the Willis-like coefficient has an interaction term between monopolar and dipolar material properties. This is consistent with the findings of Sieck *et al*. [29] showing that the Willis coupling originates from the interaction between monopole and dipole motion from asymmetries at the micro-scale. The collection of effective material properties $K_{\text{M}}^{\text{(eff)}}\left(\omega \right)$ , $\rho_{\text{M}}^{\text{(eff)}}\left(\omega \right)$ and ${\vec{S_{\text{M}}}}^{\text{(eff)}}\left(\omega \right)$ are analogous to the polarisability tensor of an individual scatterer described in [54–56]. Appendix B shows the Willis coupling coefficient of an asymmetric unit cell to illustrate the framework’s capabilities.

**Figure 6.**
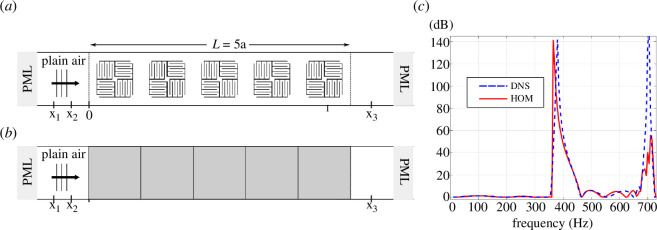
(*a*) Full-field model constituted of rigid solid labyrinthine structures (black) immersed in a plain air domain (white). (*b*) The equivalent macroscopic model where the metamaterial layer is considered as a homogenized continuum (grey) coupled to a plain air domain (white). The macro-scale mesh is shown only in the homogenized domain. (*c*) The transmission loss curve was computed via the three-microphone method for models (*a*) and (*b*).

Next, the homogenized continuum’s response in a finite-sized domain is evaluated on the configuration depicted in figure 6. A plane wave is excited at the left of the air acoustic domain. The wave enters the metamaterial layer composed of 5 unit cells sequentially arranged along the horizontal direction. The infinite character in the vertical direction is achieved by defining periodic boundary conditions on the top and bottom surfaces. Perfectly matched layer (PML) domains are employed to simulate infinitely long incoming and outgoing acoustic domains in the horizontal direction. The reference direct numerical simulation solves the full-field acoustic problem, as shown in figure 6*a*.

The homogenized response is computed using the model illustrated in figure 6*b*, combining a fluid (acoustic) domain in a $p_{a}$ -formulation in white colour coupled to the homogenized enriched continuum domain in a $\left(p_{\text{M}},{\underset{∼}{\eta }}_{\text{ɴ}}\right)$ -formulation in grey colour, implemented in COMSOL using the weak form PDE interface in the Mathematics module. The subscript a denotes the plain air domain, which is not homogenized and has material properties $\rho_{\text{a}}=\rho_{\text{f}}$ and $K_{\text{a}}=K_{\text{f}}$ . The coupling between the enriched continuum and the plain air domain is established by imposing continuity of pressure and continuity of normal acceleration, as discussed in Allard & Atalla [57] for porous materials


(4.3) $$p_{\text{M}}=p_{\text{a}}\;\text{and}\;\phi {\overset{¨}{\vec{U}}}_{\text{M}}\cdot \vec{n}=-\frac{1}{\rho_{\text{a}}}\vec{\nabla }p_{\text{a}}\cdot \vec{n},$$

where $\vec{n}$ represents the normal to the interface between the enriched continuum and the plain air. The mesh of the enriched continuum, depicted in figure 6*b*, consists of quadratic serendipity elements with a size equal to the unit cell size, i.e. 5 elements. The number of degrees of freedom for the full-field model depicted in figure 6*a* was approx. 40 000, while the model including the homogenized continuum of figure 6*b* had around 350 degrees of freedom, representing a reduction of approx. 99.1%.

To reduce computational costs, time-harmonicity of the primary fields is assumed, i.e. the simulations are performed in the frequency domain (steady-state). However, it should be stressed that the proposed framework—the initial boundary value problem (2.1), (3.30) and (3.31)—can equally be used to solve transient problems on arbitrary geometry in the time domain, i.e. problems that are not necessarily a three-medium transmission as considered here for illustration purposes. Following the approach by Lewińska *et al*. [50,58], a three-microphone method is employed to compute the transmission loss through the metamaterial layer. For a given excitation frequency ω , the complex pressure amplitudes $p_{1}$ , $p_{2}$ and $p_{3}$ are obtained as the pressure $p_{a}(\vec{x},\omega )$ averaged along the vertical lines at positions $x_{1}$ , $x_{2}$ and $x_{3}$ , as indicated in figure 6*a*,*b* . The reflection and transmission coefficients are calculated, and the transmission loss is determined using the expressions:


(4.4) $$\begin{array}{ccc} & \begin{array}{c} \begin{array}{cc} \left\{ \begin{array}{cc} R(\omega ) & =\frac{p_{2}e^{-ik_{a}x_{1}}-p_{1}e^{-ik_{a}x_{2}}}{p_{1}e^{ik_{a}x_{2}}-p_{2}e^{ik_{a}x_{1}}},\\ T(\omega ) & =\frac{p_{3}\left(e^{-ik_{a}x_{2}}+R(\omega )e^{ik_{a}x_{2}}\right)}{p_{2}e^{-ik_{a}x_{3}}}e^{-ik_{a}L} \end{array}\right.\;\;\text{and}\;\;\mathrm{TL}(\omega ) & =20\;\log_{10}\left(\frac{1}{\|T(\omega )\|}\right). \end{array} \end{array} & \end{array}$$

Here, L=5a is the total layer thickness, and $k_{a}=\omega /\sqrt{K_{a}/\rho_{a}}$ is the wavenumber in the horizontal direction in the plain air acoustic domain.

The transmission losses, computed with the direct numerical simulation and the homogenized enriched continuum, are compared in figure 6*c*. An excellent agreement is observed up to approx. 550 Hz. However, a discrepancy between the homogenized continuum and the reference solution is evident for higher frequencies from approx. 350 Hz onwards, as also observed in the dispersion analysis (figure 5). The underestimation of the sound speed shown in figure 5 correlates with the underestimation of the transmission loss peak due to resonance in figure 6. This can be attributed to the decomposition (3.8), which is no longer valid for a short wavelength with respect to the unit cell size, including the largest peak due to the negative bulk modulus effect. A study on the influence of the macro-scale mesh has been performed (not shown here). It has been found that for a metamaterial with a macro-scale mesh with more than five elements, the transmission curve remains practically unchanged. On the other hand, a significant error is observed for fewer than five elements, which is to be expected since, in this case, the effective wave is not accurately discretized.

As evident from the above results, the framework is valid for (low) frequencies—up to approx. 550 Hz in the considered case—that satisfy the scale separation at the unit cell boundary, i.e. the non-coiled region, which can be referred to as the *host* domain. Therefore, the effectiveness of the framework for accurately upscaling the micro-scale dynamics relies on the subwavelength character of the resonance, which is given by the contrast between the *host* domain (non-coiled) and the *resonant* domain (the labyrinth). For the considered unit cell, there is a geometric contrast of approx. 7 times, whereas, for instance, in the unit cell of Liu *et al*. [1], there is a material contrast of 1000 in stiffness between the *host* matrix and the *resonant* rubber-coated inclusion, ensuring a highly localized (subwavelength) resonance. To conclude, high contrast between *host* and *resonant* domains leads to a unit cell with more locally resonant modes that can be upscaled with the present framework. Here, a relatively low contrast is analysed, and it illustrates well the limit of the hypothesis on the validity of the equation (3.8), which is satisfied only by the first mode in this case.

## 5. Conclusion

The high computational cost for finite-size metamaterial simulations arises from its fine geometric features. Reduced-order computational homogenization is a solution to address this issue. This article proposes an efficient multiscale model for analysing the transient response of acoustic LRAMs based on a pressure formulation and it is applicable to both frequency- and time-domain analysis. The method couples macro- and micro-scales using an extended version of the Hill-Mandel principle. The micro-scale dynamics are represented in a reduced space as the superposition of long wavelength and local resonance responses. The outcome is a macroscopic homogenized enriched continuum with additional enrichment variables describing the micro-scale dynamics in a subwavelength regime. The enrichment variables may be eliminated if a frequency domain analysis is performed. The upscaled effective constitutive model is versatile, modelling exotic macroscopic responses typically found in acoustic metamaterials. The dispersion relation and the considered finite-size problem indicate that the homogenized model captures the behaviour of the direct numerical simulations very well within the limits of its applicability, demonstrating the framework’s efficacy and accuracy.

Other versatile results can be obtained with the presented framework. For example, a thorough investigation of the unit cell symmetries and their consequences on the homogenized coefficients for an LRAM unit cell with non-negligible Willis coupling. Another result is a straightforward extension of the current framework considering a deformable solid internal to the unit cell, i.e. disconnected solid structures such as the unit cell of this article. A solid and fluid resonance can be designed to take place in a close frequency range, yielding interesting metamaterial behaviour originating from co-existing subwavelength resonances. Notably, in the latter case, the macro-scale model remains the same as presented here: an equivalent fluid supporting only compression waves since the solid phase is not connected.

The approach developed in this article will facilitate the design of metamaterials in realistic industrial scenarios where fast simulations are required for optimization. Therefore, the proposed homogenization framework is an important step to accelerate progress towards achieving a higher *Technology Readiness Level* for the emerging class of metamaterials, where numerous applications await exploration of their full design space, as pointed out by Kochmann *et al*. [59]. Moreover, this framework paves the way for developing multiscale methods for high-fidelity acoustic problems, where more elaborate material models are required, including dissipation and flow-induced nonlinearities; for instance, acoustic liner applications, as discussed in Jones *et al*. [60] and Rego *et al*. [61].

## Figures and Tables

**Figure 7.**
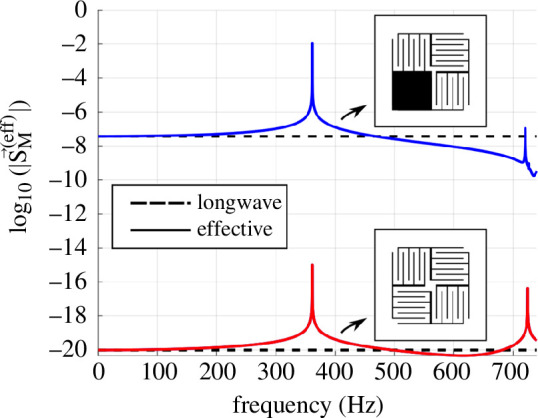
The absolute value of the effective Willis coupling coefficient obtained via the computational homogenization approach for a symmetric unit cell plotted as a solid red line and an asymmetric unit cell plotted as a solid blue line. The long wavelength limits are dashed black lines. Interpretation of curve's colors in the figure is not necessary since the corresponding unit cell is indicated with an arrow.

## Data Availability

The code (MATLAB script) and COMSOL models supporting this article can be found at 4TU.ResearchData repository [62].

## Appendix A: Evolution equations and macroscopic constitutive relations

Inserting equation (3.29) in equation (3.15) gives

(A 1)
$$\begin{array}{rl} {\underline{I}}_{\text{ɴɴ}}^{\left(lr\right)}\;{\underset{∼}{\ddot{\eta }}}_{\text{ɴ}}+{\underline{\Lambda }}_{\text{ɴɴ}}^{\left(lr\right)}\;{\underset{∼}{\ddot{\eta }}}_{ɴ} & =-{\underline{m}}_{\text{ɴʙ}}^{\left(c\right)}\;{\underset{∼}{\ddot{p}}}_{\text{ʙ}}\\ & =-{\underline{m}}_{\text{ɴʙ}}^{\left(c\right)}\;\left({\underset{∼}{1}}_{\text{ʙ}}\;{\ddot{p}}_{M}+\vec{\nabla_{M}}{\ddot{p}}_{M}\cdot \Delta {\underset{∼}{\vec{x}}}_{\text{ʙ}}\right)\\ & =\overset{\underset{∼}{\gamma }\equiv }{\overbrace{\left[-{\underline{m}}_{\text{ɴʙ}}^{\left(c\right)}\;{\underset{∼}{1}}_{\text{ʙ}}\right]}}\;{\ddot{p}}_{M}+\overset{\underset{∼}{\vec{\delta }}\equiv }{\overbrace{\left[-{\underline{m}}_{\text{ɴʙ}}^{\left(c\right)}\;\Delta {\underset{∼}{\vec{x}}}_{\text{ʙ}}\right]}}\cdot \vec{\nabla_{M}}{\ddot{p}}_{M} \end{array}$$

Since the left-hand side matrices in the above equation are diagonal, it can be written in the form of the evolution equations (3.31) for each mode j separately:

(A 2)
$$\overset{¨}{\eta_{j}}+\;\;\omega_{j}^{2}\eta_{j}\;=\;\;\;\gamma_{j}\;\;{\overset{¨}{p}}_{\text{M}}\;\;\;\;+\;\;\;\vec{\delta_{j}}\cdot \vec{\nabla_{\text{M}}}{\overset{¨}{p}}_{\text{M}}\;\;\text{for eigenmodes}\;\;j=1,2,...,\text{ɴ}.$$

Equation (3.16) is used for computing the external micro flux ${\underset{\sim }{q}}_{\text{ʙ}}^{\text{ ext}}$ that can be found by inserting equation (3.29) into (3.16). After this step, ${\underset{\sim }{q}}_{\text{ʙ}}^{\text{ ext}}$ is inserted in the discretized equations (3.28), providing the closed-form constitutive relations for the volumetric strain rate

(A 3)
$$\begin{array}{ll} \phi {\ddot{\epsilon }}_{M}= & \overset{C_{\epsilon }\equiv }{\overbrace{[-\frac{1}{V}{\underset{∼}{1}}_{\text{ʙ}}^{T}\;\;{\underline{Q}}_{\text{ʙʙ}}\;\;{\underset{∼}{1}}_{\text{ʙ}}]}}{\ddot{p}}_{M}+\overset{\vec{D_{\epsilon }}\equiv }{\overbrace{[-\frac{1}{V}{\underset{∼}{1}}_{\text{ʙ}}^{T}\;\;{\underline{Q}}_{\text{ʙʙ}}\;\;\Delta {\underset{∼}{\vec{x}}}_{\text{ʙ}}]}}\cdot \vec{\nabla_{M}}{\ddot{p}}_{M}\\ + & \overset{G_{\epsilon }\equiv }{\overbrace{[-\frac{1}{V}{\underset{∼}{1}}_{\text{ʙ}}^{T}\;\;{\underline{H}}_{\text{ʙʙ}}\;\;{\underset{∼}{1}}_{\text{ʙ}}]}}p_{M}+\overset{\vec{H_{\epsilon }}\equiv }{\overbrace{[-\frac{1}{V}{\underset{∼}{1}}_{\text{ʙ}}^{T}\;\;{\underline{H}}_{\text{ʙʙ}}\;\;\Delta {\underset{∼}{\vec{x}}}_{\text{ʙ}}]}}\cdot \vec{\nabla_{M}}p_{M}\\ + & \overset{{\underset{∼}{c}}^{T}\equiv }{\overbrace{[-\frac{1}{V}{\underset{∼}{1}}_{\text{ʙ}}^{T}\;\;{\underline{m}}_{\text{ʙ}\text{ɴ}}\;\;]}}{\ddot{\underset{∼}{\eta }}}_{\text{ɴ}} \end{array}$$

and for the fluid acceleration

(A 4)
$$\begin{array}{rl} \phi {\ddot{\vec{U}}}_{M}= & \overset{\vec{C_{U}}\equiv }{\overbrace{[-\frac{1}{V}\Delta {\underset{∼}{\vec{x}}}_{\text{ʙ}}^{T}\;\;{\underline{Q}}_{\text{ʙ}\text{ʙ}}\;\;{\underset{∼}{1}}_{\text{ʙ}}]}}{\ddot{p}}_{M}+\overset{D_{U}\equiv }{\overbrace{[-\frac{1}{V}\Delta {\underset{∼}{\vec{x}}}_{\text{ʙ}}^{T}⊗\;\;{\underline{Q}}_{\text{ʙ}\text{ʙ}}\;\;\Delta {\underset{∼}{\vec{x}}}_{\text{ʙ}}]}}\cdot {\vec{\nabla }}_{M}{\ddot{p}}_{M}\\ + & \overset{\vec{G_{U}}\equiv }{\overbrace{[-\frac{1}{V}\Delta {\underset{∼}{\vec{x}}}_{\text{ʙ}}^{T}\;\;{\underline{H}}_{\text{ʙ}\text{ʙ}}\;\;{\underset{∼}{1}}_{\text{ʙ}}]}}p_{M}+\overset{H_{U}\equiv }{\overbrace{[-\frac{1}{V}\Delta {\underset{∼}{\vec{x}}}_{\text{ʙ}}^{T}⊗\;\;{\underline{H}}_{\text{ʙ}\text{ʙ}}\;\;\Delta {\underset{∼}{\vec{x}}}_{\text{ʙ}}]}}\cdot {\vec{\nabla }}_{M}p_{M}\\ + & \overset{{\underset{∼}{d}}^{T}\equiv }{\overbrace{[-\frac{1}{V}{\underline{m}}_{\text{ʙ}\text{ɴ}}^{T}\;\;\Delta {\underset{∼}{\vec{x}}}_{\text{ʙ}}{]}^{T}}}{\ddot{\underset{∼}{\eta }}}_{\text{ɴ}} \end{array}$$

or, in short, equation (3.30).

The coefficients $G_{\epsilon }$ , $\vec{H_{\epsilon }}$ and $\vec{G_{U}}$ depend on the product $H_{\text{ʙ}\text{ʙ}}\;{\underset{\sim }{1}}_{\text{ʙ}}$ , vanishing identically, since ${\underset{\sim }{1}}_{\text{ʙ}}$ belongs to the null space of $H_{\text{ʙ}\text{ʙ}}$ . Mathematically analogous to the solid rigid body motion, this corresponds to the case when the fluid undergoes a homogeneous change in pressure ${\underset{\sim }{1}}_{\text{ʙ}}$ on the prescribed nodes ʙ , in the absence of flow, in other words, a homogeneous volume expansion/contraction takes place. Neglecting the identically zero coefficients, the eigenvalue problem reads

(A 5)
$$\left(\left(-\omega^{2}\right)\left[\begin{array}{ll} C_{\epsilon }+\vec{k}\cdot D_{U}\cdot \vec{k}+i(-\vec{D_{\epsilon }}\cdot \vec{k}+\vec{k}\cdot \vec{C_{U}}) & \;(\underset{∼}{c}+i\vec{k}\cdot \underset{∼}{\vec{d}}{)}^{T}\\ -\underset{∼}{\gamma }+i\vec{k}\cdot \underset{∼}{\vec{\delta }} & \;{\underline{I}}_{\text{ɴ}\text{ɴ}}^{\text{(lr)}} \end{array}\right]+\left[\begin{array}{ll} \vec{k}\cdot H_{U}\cdot \vec{k} & \;{\underset{∼}{0}}_{\text{ɴ}}^{T}\\ {\underset{∼}{0}}_{\text{ɴ}} & \;{\underline{\Lambda }}_{\text{ɴ}\text{ɴ}}^{\text{(lr)}} \end{array}\right]\right)\left(\begin{array}{l} {\hat{p}}_{\text{M}}\\ {\underset{∼}{\hat{\eta }}}_{\text{ɴ}} \end{array}\right)=\left(\begin{array}{l} 0\\ {\underset{∼}{0}}_{\text{ɴ}} \end{array}\right).$$

The motivation for the choice of $\vec{x_{R}}$ is made such that certain coefficients are significantly reduced, for example, ${𝐃}_{U}$ and ( $\vec{D_{\epsilon }}$ , $\vec{C_{U}}$ ), in which the latter pair becomes zero in the case of a square symmetric unit cell. Note that even in the case ( $\vec{D_{\epsilon }}$ , $\vec{C_{U}}$ ) are nonzero, for an asymmetric unit cell, the sum $\left(-\vec{D_{\epsilon }}\cdot \vec{k}+\vec{k}\cdot \vec{C_{U}}\right)$ vanishes in the diagonal of matrix (A 5). This follows from the equality of the coefficients $\vec{D_{\epsilon }}=\vec{C_{U}}$ since ${\underline{Q}}_{\text{ʙ}\text{ʙ}}$ is a symmetric matrix (see equations (A 3) and (A 4) ). It, therefore, preserves the self-adjointness of the operator on the state vector ${\left[{\hat{p}}_{\text{M}}\;{\underset{∼}{\hat{\eta }}}_{\text{ɴ}}\right]}^{\text{T}}$ that leads to an eigenproblem with real eigenvalues, which is expected for a conservative system. Logically, the eigenvalue problem of the homogenized continuum has real eigenfrequencies since the full-field response does not decay with time, which aligns with the results of Willis [63] showing that self-adjointness at the micro-scale implies self-adjointness at the macro-scale.

## Appendix B: Willis coupling for asymmetric unit cell

The effective Willis coupling coefficient ${\vec{S_{\text{M}}}}^{\text{(eff)}}\left(\omega \right)$ , defined in equation (4.2) for the symmetric unit cell, figure 3, is shown in figure 7. A new unit cell with a prominent asymmetry was created by replacing a quarter of the labyrinth domain with a rigid solid block, as shown in figure 7. The asymmetric unit cell has a Willis coupling coefficient that is several orders of magnitude larger than the symmetric unit cell, even in the long wavelength limit.

The asymmetric unit cell has monopolar material properties $c_{1}=1.192\times 10^{-2}$ approximately the same as the symmetric one (table 1). The dipolar material property, on the other hand, is $\vec{d_{1}}=-\left(0.1590\;\vec{e_{1}}+0.1657\;\vec{e_{2}}\right)\times 10^{-3}$ m, which is large compared with the symmetric unit cell where $\vec{d_{1}}=\vec{0}$ , see table 1. The break in symmetry induces a non-negligible dipolar resonance $\vec{d_{1}}$ for the first mode that interacts with the monopolar resonance $c_{1}$ , leading to a larger Willis coupling, as expected from equation (4.2).
